# Insufficient Sleep and Alzheimer’s Disease: Potential Approach for Therapeutic Treatment Methods

**DOI:** 10.3390/brainsci15010021

**Published:** 2024-12-28

**Authors:** Dieu Quynh Trinh, Nhu Huynh Mai, Toan Duc Pham

**Affiliations:** 1 Faculty of Applied Sciences, Ton Duc Thang University, Ho Chi Minh City 700000, Vietnam; trinhquynhdieu@tdtu.edu.vn; 2 Faculty of Pharmacy, University of Medicine and Pharmacy at Ho Chi Minh City, Ho Chi Minh City 700000, Vietnam; mhnhu@ump.edu.vn; 3 Faculty of Pharmacy, Ton Duc Thang University, Ho Chi Minh City 700000, Vietnam

**Keywords:** Alzheimer’s disease, sleep deprivation, aging, neuroinflammation

## Abstract

The interaction between Alzheimer’s disease (AD) and sleep deprivation has recently gained attention in the scientific literature, and recent advances suggest that AD epidemiology management should coincide with the management of sleeping disorders. This review focuses on the aspects of the mechanisms underlying the link between AD and insufficient sleep with progressing age. We also provide information which could serve as evidence for future treatments of AD from the early stages in connection with sleep disorder medication.

## 1. Introduction

Sleep is a normal physiological activity for many creatures on Earth. For most people, sleep is a very simple concept: they believe that the brain enters a resting state and stops working during the process of sleeping. However, studies have demonstrated that our brain and body actually perform functions during the sleeping period. It turns out that the brain activity that occurs during sleep is as important as that while awake. Sufficient sleep has indispensable functions in terms of energy saving, cell repair, thermoregulation and metabolic homeostasis [1]. Björn Rasch and Jan Born suggested that different functions of sleep are implicated in memory-related processes, such as the reduction of oxidative toxicity and glycogen replacement [1]. The physiology of sleep is connected with the physiology of memory through the consolidation of systems. Different points of view have been presented to explain the role of sleep in systems consolidation processes. The first one focuses on the reactivation of hippocampal neurons during sleep and its correlation with the formation of long-term memory. This point of view is supported by the evaluation of firing rates and firing patterns of neurons, as well as indirect evidence obtained in functional magnetic resonance imaging (fMRI) and electroencephalogram (EEG) studies [2]. The second point of view focuses on the selective downregulation of rarely activated synapses or those less fitted with previous consolidated memories. Only synapses with strong activation and connectivity with old memories remain, survive, and are gradually consolidated [3]. Systems consolidation, unlike simple synaptic consolidation, can last for months or even years. Thus, impairment associated with systems consolidation during sleep is a progressive process. Therefore, it is plausible that the mechanisms underlying sleep disorders could affect memory consolidation in the context of a progressive disorder such as Alzheimer’s disease.

Sleep is divided into two major phases: non-rapid eye movement (NREM) and rapid eye movement (REM) sleep [4]. Deep non-rapid eye movement sleep, or slow wave sleep (SWS), is well recognized for its role in the restoration of brain function [5]. REM sleep is believed to be the time in which our brain generates long-term memory through an active systems consolidation process [6,7,8]. During the REM sleep period, memories that are temporarily stored in the hippocampus are repeatedly reactivated, inducing neuroplastic changes in the cortical areas which result in the formation of long-term memories [9,10]. The beneficial action of sleep on memory requires the precise regulation of neurotransmitter, endocrine and immunological factors [1]. Thus, dysfunction in the active process of sleep can potentially result in neuropsychiatric disorders.

Alzheimer’s disease (AD) is a common progressive neurodegenerative disease that affects millions of people worldwide [11]. AD causes a decline in memory function, learning ability and cognitive deficits in elderly people. Symptomatic AD starts after the onset of impairment in cognitive profiles. However, the biological mechanism of AD becomes visible 15–20 years before the onset of cognitive impairment [12]. The pathology of AD is characterized by the appearance of amyloid and tau proteins in the brain [13,14]. The quality of sleep decreases gradually as humans age, and insufficient sleep is considered a risk factor for AD. On the other hand, neurodegeneration in the context of AD injures sleep-regulated brain regions such as the hypothalamus, where the ventrolateral preoptic area (VLPO) is located. Therefore, the relationship between sleep disorders and AD is thought to be bidirectional [15]. It is well known that AD-related neurodegeneration accumulates for 15–20 years before patients present cognitive dysfunction and memory impairment [15]. The AD pathology and sleep disorders may share common progressive characteristics, with sleep disorders and AD both worsening with age. Both disorders are strongly connected with biological and neurological changes throughout aging. Thus, understanding the mechanisms underlying the connection between the progress of AD and sleep disorders with age may reveal new therapeutic treatments for the control of AD pathogenesis.

## 2. Sleep Architecture Alterations and Alzheimer’s Disease Pathogenesis

Patients with AD often suffer from symptoms of sleep deprivation, with prevalence rates around 71% [16]. Sleep disturbance symptoms in AD are manifested as changes in the sleep/wake cycle, such as increasing night-time awakenings and fragmented sleep [17,18]. AD also induces changes in sleep architecture, significantly decreasing SWS and REM sleep, which are responsible for memory consolidation, neurogenesis and neuronal repair [19,20,21]. However, changes in the sleep of patients with AD is not just a consequence of the disease but could be the causative factor that induced the progression of AD from the very early stages. Studies have reported that sleep disturbances may precede the appearances of other AD-related cognitive symptoms and increase the risk of developing AD pathology [17,22,23,24]. Sleep fragmentation, measured via actigraphy, increased the probability of developing AD in elderly people [25]. Sleep deprivation exacerbated the severity of dementia behaviors and AD pathological markers in an animal model of AD [26,27]. Suvorexant—an insomnia medication—has been reported to ameliorate cognitive impairments and the pathology of AD in both preclinical and clinical studies [28,29,30]. AD is a progressive disorder, meaning that its symptoms gradually aggravate over many years [31]. Early diagnosis and treatment of AD have shown marked benefits in terms of economic efficiency and cost saving [32,33]. The exhibition of specific oscillatory patterns of sleep in patients with sleep disturbances may serve as a tool for the early diagnosis of AD.

## 3. Lack of Sleep Impacts Beta-Amyloid (Aβ) Levels

In the pathogenesis of AD, Aβ accumulation is known as a cause of synaptic dysfunction and neuronal apoptosis [34]. Aβ accumulation occurs in the early stage of AD and is followed by other changes in biomarkers of AD, such as synaptic dysfunction, Tau-mediated neuronal injury, and cognitive impairments [35]. Physiological investigation of Aβ has established that cerebrospinal fluid (CSF) Aβ levels can be used as a surrogate marker for amyloid plaque aggregation in clinical studies [36]. Moreover, Aβ levels in CSF showed rising and falling patterns depending on fluctuations in the circadian rhythm. Aβ levels in CSF and in the brain are mediated by the balance between its production and clearance [37,38]. Therefore, it is plausible that an alteration in the sleeping period could induce an imbalance in Aβ metabolism and increase the Aβ level, thus contributing to the development of AD. Numerous animal studies have also clarified the role of insufficient sleep on the increase in Aβ level. A sleep deprivation rat model showed impairment in cognitive function correlated with a significant increase in Aβ peptides. Changes in the level of Aβ peptides have been implicated with an imbalance in Aβ metabolism, which potentiates β-secretase levels, while Aβ-degradation enzymes remain unchanged [39]. A chronic lack of sleep has been shown to accelerate Aβ aggregation, while pharmacological recovery of the sleeping period rescued mice from the accumulation of Aβ [40]. Sleep- restricted mice have been shown to present increased Aβ oligomer protein expression and accentuated memory deficits, as assessed through a contextual memory test [41]. Neuronal activity during wakefulness has been implicated in increased Aβ levels [42,43]. On the other hand, more sleeping time potentiates the glymphatic system, which plays a functional role in amyloid clearance in the brain. In addition, astrocytic activity has been reported to regulate the glymphatic system in a circadian manner [44]. Astrocytes are known for their protective effects against neurodegenerative disease. Astrocytic activity maintains the homeostasis of the microenvironment around neurons, and astrocytes are a key component of the BBB, regulating the substances that travel in and out of the brain. Astrocytic excitatory amino acid transporters (EAATs) uptake glutamate at synapses. Astrocytes also preserve the bicarbonate extracellular buffer of the brain through providing HCO_3_^−^. Regulation of astrocytic glutathione peroxidase-1 via the JAK2/STAT3 signaling pathway attenuated neuronal apoptosis and neuroinflammation [45,46]. In a case study, the daily rhythm of astrocytic water channel aquaporin-4 (AQP4) vascular polarization to the perivascular endfeet regulated the glymphatic rhythm in the brain, supporting homeostasis. The circadian clock gene *Arntl1* mediates the rhythmic localization of AQP4 to the perivascular endfeet of astrocytes. Chronic sleep disruption could decrease AQP4 polarization, leading to glymphatic clearance dysfunction and accumulation of Aβ [47,48]. Therefore, it is plausible that insufficient sleep can also downregulate the protective effects of astrocytes and that the equilibrium of astrocytic clock genes is important in preventing Aβ accumulation as well as AD pathogenesis.

## 4. Lack of Sleep Impacts Tau Aggregation

Tau is a normal protein in the brain, which plays the physiological role of stabilizing neuronal microtubules [49]. However, pathological Tau aggregation has been observed in many neurodegenerative diseases, including AD. Tau aggregation induced the progress of formation of neurofibrillary tangles (NFTs). Many elements are responsible for the tau aggregation process, such as *MAPT* gene mutation, dysfunction in post-translational modification or misfolded protein formation [50]. While phosphorylation is pivotal for the normal physiological function of Tau, hyperphosphorylation may induce the pathological properties [51]. Sleep deprivation can interfere with synaptic phosphorylation rhythms in the brain [52]. It has been found that sleep deprivation increased both unphosphorylated and phosphorylated Tau [53,54]. These results suggest that a lack of sleep can accentuate the severity of Tau aggregation in patients with AD. Animal studies have also indicated the interconnection between Tau protein and sleep disturbance. PS19 Tau transgenic significantly altered the sleep architecture, including decreased REM and non-REM sleep [55]. ADRB1 is a receptor located on noradrenergic (NE) neurons in the locus coeruleus (LC). These noradrenergic neurons function in regulating the sleep–wake cycle [56]. NE oscillations controlled by these neurons improve memory consolidation through altering REM and non-REM sleep patterns [57]. LC neurons are also among the earliest neurons to express AD-like pathological changes, including Tau hyperphosphorylation [58]. Using a PS19 mouse model with Adrb1-A187V mutation, Qing Dong et al. observed that Adrb1-A187V;PS19 mice showed less profound Tau aggregation, Tau hyperphosphorylation, and REM sleep reduction in comparison with PS19 mice. Moreover, the Adrb1-A187V mutation reversed sleep deprivation causing memory loss in PS19 mice [56]. Taken together, lack of sleep has a strong connection with Tau aggregation, and recent studies have achieved positive results in terms of clarifying the molecular mechanisms through which sleep induces Tau aggregation in AD pathophysiology.

## 5. Lack of Sleep Impacts Oxidative Stress Related to AD

Oxidative stress plays a pivotal role in neurodegeneration and cognitive dysfunction in patients with AD. Oxidative stress induces DNA damage, mitochondrial dysfunction, and the formation of neurotoxic substances, contributing to the pathogenesis of AD [59,60,61]. Mitochondria are a major source of reactive oxygen species (ROS) [62]. As mitochondrial respiration exhibits circadian oscillation, oxidative status follows the same pattern. Prior research has revealed that night shift workers have deficits in antioxidant enzyme systems and higher levels of reactive oxygen species (ROS) than day workers [63]. According to the establishment that characteristics of sleep in the fruit fly *Drosophila melanogaster* share common features with mammalian sleep, researchers have conducted experiments to evaluate the effects of sleep deprivation on the induction of oxidative stress-related damages [64]. Kyunghee Koh et al. reported that oxidative stress related to aging can induce sleep fragmentation [65]. The induction of ROS is also reported to be correlated with sleep deprivation. An antioxidant drug treatment was able to rescue sleep deprivation-induced death in a fruit fly model [66]. Therefore, sleep loss could cause oxidative stress and thus cause damage to the brain. Studies conducted on rodents have also indicated the effects of sleep deprivation on decreased glutathione levels and superoxide dismutase (SOD) activity [67,68]. The glutathione system and SOD have been shown to protect against neurodegeneration in animal models of AD [69,70]. Taking these results together, insufficient sleep-induced oxidative stress can be considered as a target for the development of therapeutic approaches for the treatment of AD.

## 6. Lack of Sleep Impacts Neuroinflammation Related to AD

The pathology of AD is characterized by the induction of Aβ and Tau aggregation. However, AD’s pathological physiology is not only related to these protein dysfunctions but also includes several dysregulations in brain functional systems. One of the important potential factors in the pathogenesis of AD is neuroinflammatory processes [71]. Neuroinflammation in the brain consists of the secretion of inflammatory cytokines and the activation of inflammatory microglia and astrocytes. Higher levels of C-reactive protein (CRP), a marker of inflammation, have been correlated with more sleep fragmentation and high risk of dementia [72]. Interleukin-6 (IL-6) has been associated with the interaction between AD and the apnea–hypopnea index (AHI) [73]. Studies in animals have also shown that sleep deprivation causing neurodegeneration and memory impairments associated with microglia-modulated neuroinflammation [74,75,76,77]. Sleep fragmentation has been associated with microglial aging and activation, which are causative factors of cognitive impairment in AD [78]. Normal sleep facilitates astrocytic adenosinergic A1, A2, and A3, suppressing neuronal overactivity, which can lead to increases in Aβ and Tau accumulation. In contrast, sleep disturbances drive the activation of inflammatory microglia and astrocytes, thus releasing inflammatory cytokines which induce Aβ and Tau aggregation [79]. Interestingly, neuroinflammation also mediates neurogenesis in both beneficial and detrimental manners. Inflammatory IL-1β and IL-6 inhibit hippocampal neurogenesis [80,81]. On the other hand, mild acute inflammation potentiates neurogenesis [82]. Hippocampus samples from patients with AD were found to express more neurogenesis marker proteins [83]. Sleep deprivation has been shown to cause inhibition of adult neurogenesis [84,85,86,87,88]. Therefore, insufficient sleep seems to break the immunological homeostasis that inhibits AD pathophysiology. There is initial evidence that suggests the role of sleep in regulating the immune system in the AD brain. Sleep deprivation impairs spatial memory and inhibits hippocampal neurogenesis through inducing an imbalance in inflammatory cytokines and the activation of inflammatory microglial cells [77].

## 7. Lack of Sleep Impacts the Expression of Clock Genes Related to AD

Sleep disturbance is just one of a bundle of circadian process impairments implicated in the pathology of AD. These impairments include alterations in the endocrine system, blood pressure, and enzymatic synthesis. Circadian rhythm dysfunction is thus considered a common symptom of AD [89]. The circadian rhythm is regulated by the activation and deactivation of clock genes. Sleep loss can interfere with the homeostatic mechanism of clock genes related to stress induction [90]. Sleep deprivation can lead to a decrease in DNA binding of clock to the *Dbp* gene, as well as Bmal1 and Npas2 to the *Dbp* and *Per2* genes [91]. The depletion of Bmal1, clock, and Npas2 impaired hippocampus-dependent learning [92,93,94]. Therefore, a lack of sleep could alter the functions of hippocampus learning and memory storage. Retrosplenial cortex (RSC) neurons showed abnormal Bmal1 expression co-localized with phosphorylated Tau (p-Tau) [95]. Global Bmal1 depletion increases ApoE expression and Aβ plaque deposition [96]. These results provide evidence that link the circadian impairment caused by sleep loss to the development of AD neuropathology. Melatonin is an important hormone synthesized by the pineal gland, which is released in response to darkness [97]. The expression of clock genes is modulated by the secretion of melatonin [98,99]. Melatonin has been used for treating patients with circadian problems such as jet lag and misaligned circadian rhythm in shift workers. Melatonin also improves quality of sleep and restores the suprachiasmatic circadian rhythm [100,101]. Treatment with melatonin has been reported to have protective effects against memory impairment, neurodegeneration, oxidative stress, and neuroinflammation [102,103,104,105,106]. Melatonin also attenuated the pathophysiology of AD, including attenuation of amyloid deposition, neuronal apoptosis, and cholinergic impairment, in an animal model of AD [107]. In patients with AD, melatonin levels are decreased compared with those of normal persons [108]. Many studies have indicated that sleep deprivation alters the melatonin rhythmic system and may disrupt the compensative mechanism of melatonin, thus causing circadian-related symptoms in patients with AD [109,110].

## 8. Sleeping Disorder Therapeutic Treatment in Association with AD Pathology

As we suspect that insufficient sleep contributes to AD pathology, preclinical AD pathology treatment should include sleeping management methodology. Treatment strategies for patients suffering from insufficient sleep can involve non-pharmacological and pharmacological approaches.

### 8.1. Non-Pharmacological Approaches

Photobiomodulation (PBM) is one of the non-pharmacological methods that can be applied to manage sleep quality in association with biochemical changes in patients with AD [111]. PBM can regulate melatonin biosynthesis, thus recovering the circadian rhythms [112]. Improvements in sleep quality are reported to be beneficial for patients with AD [113,114]. PBM can reduce neuropathologies such as Aβ production in the hippocampus and microglial activation and can attenuate cognitive dysfunction associated with AD [115,116]. However, one systematic review has indicated that light therapy effectiveness may have been interfered with by pathology differences in specific research [111]. Therefore, deeper investigation into the effects of PBM on biological changes during AD neuropathogenesis in an APP and PSEN1 transgenic mouse model could provide further clues in the search for better markers with which to design more consistent clinical trials.

Repetitive transcranial magnetic stimulation (rTMS) is another non-pharmacological neuromodulatory method for the treatment of sleep disorders and neurodegenerative diseases [117,118]. rTMS is a technique which uses magnetic fields to stimulate neuron activity to improve mood and cognitive conditions. For AD treatment, rTMS targets the dorsolateral prefrontal cortex (DLPFC), either laterally or bilaterally, and ameliorates cognitive dysfunction [119,120,121,122]. rTSM has also shown positive results in the treatment of insomnia [123,124]. Interestingly, these studies targeted DLPFC—which is the same as the target in AD—for investigating the effect of rTSM on sleep disorders. Although the mechanism of rTSM is not yet fully understood, crosstalk between rTSM therapy for AD and sleep disorders might reveal how a therapy for insomnia could prevent AD neuropathogenesis. However, a randomized placebo-controlled double-blind clinical trial in Australia reported contrary results, i.e., that rTSM therapy indicates no significant cognitive improvement in patients with AD [125]. Hence, further studies have to be conducted to determine the reason for this controversy.

### 8.2. Pharmacological Approaches

Pharmacological treatments for patients with AD have to be considered carefully. Even though a lack of sleep can aggravate AD neuropathogenesis, using benzodiazepines (BDZs) in order to manage sleeping time could increase the risk of stroke or even hasten AD neuropathogenesis [126,127]. On the other hand, one study reported the opposite result, i.e., that BDZs treatment inhibited Aβ aggregation [128]. Z-drugs are generally assumed to be more safe for patients with AD than BDZs [129]. Taken together, there is not enough evidence to conclude that it is good or bad to use hypnotics in the case of AD. That chronic use of BDZs can increase the risk of developing AD has also been debated. The fact that insufficient sleep is thought to be early a symptom of AD makes it even more complicated. In our opinion, different points of view should be considered concerning the effects of hypnotics on AD. The pharmacological effects of BDZs and Z-drugs rely on the effects of these drugs on the GABA receptor. GABA is well known to be an inhibitory neurotransmitter which down-regulates neuronal activity. This characteristic can lead to the inhibition of neurons that need to be active during the sleep stages and can induce changes in sleep architecture. Changes in sleep architecture can bring about deficits in concentration and working memory [130]. Patients with AD at the clinical stages who suffer from insufficient sleep can received benefits from BDZs with careful prescription. However, the use of BDZs at the early and preclinical stages of AD should be avoided as much as possible.

Recently, melatonin has become a more and more familiar part of AD treatment [131]. Clinical trials have suggested that using melatonin has protective effects against AD [132,133]. The doses and duration of melatonin treatment have been studied and reviewed [134,135]. Intranasal administration of melatonin is reported to be effective in assisting sleeping. Increasing melatonin levels in the brain via intranasal administration represents a potential therapeutic approach to alleviating an AD-induced deficiency of melatonin. The relevant mechanism of melatonin is not just about making people fall asleep and extending the sleeping time; melatonin works as a circadian shield, protecting against Aβ toxicity, tau pathology, oxidative stress, BBB breakdown, and glymphatic dysfunction [136]. Thus far, therapeutic uses of melatonin have focused on its role in managing AD-related insomnia [107] and have not yet touched on the effects of melatonin on the early stages of AD. Early methods of predicting AD via artificial intelligence (AI) are being developed [137,138]. This enables researchers to investigate whether melatonin or other similar sleeping medicines effect AD pathogenesis when used as a functional food at a time when clinical AD has not manifested.

## 9. Discussion and Conclusions

AD is one of the most common neurodegenerative diseases causing dementia in older individuals. This disease affects not only patients but also the health conditions of their caregivers. The treatment of AD costs more than USD 300 billion a year [139]. Therefore, the management of AD is an important demand. Our work reviewed the connection between insufficient sleep and the development of AD neuropathy. Recent findings have suggested that the management of sleep deprivation could play a part in taking control of the epidemiology of AD. Sleep loss also disrupts the systemic circadian rhythm. Drugs that recover the circadian system, such as those with effects on melatonin and the orexin endocrine system, can provide new approaches for the management of AD from the very early stages.

Cross-talk between the medication used for sleep deprivation and AD has been disclosed in the case of melatonin. Exogenous melatonin is often used for the treatment of various sleeping problems and is considered to be safe, with fewer side effects than other sedative hypnotics [140]. A recent study demonstrated melatonin to have a potential protective effect against AD [136]. However, the protective effects of melatonin were not observed in patients with late-stage AD [136,141,142]. In many countries, including Vietnam, melatonin is considered a familiar functional food supporting patients with sleep disorders, and it is easily accessible and affordable. Therefore, pharmacological changes associated with the use of melatonin could reveal which circadian biomarkers have potential for enabling the early diagnosis of AD. In the future, the effects of melatonin as a preventive agent for pre-clinical AD should be investigated, along with clinical AD.
